# Applicability of Anti-Human Estrogen Receptor β Antibody PPZ0506 for the Immunodetection of Rodent Estrogen Receptor β Proteins

**DOI:** 10.3390/ijms20246312

**Published:** 2019-12-13

**Authors:** Hirotaka Ishii, Mai Otsuka, Moeko Kanaya, Shimpei Higo, Yujiro Hattori, Hitoshi Ozawa

**Affiliations:** 1 Department of Anatomy and Neurobiology, Graduate School of Medicine, Nippon Medical School, 1-1-5, Sendagi, Bunkyo-ku, Tokyo 113-8602, Japan; truewear0915@gmail.com (M.O.); kanayamoeko@gmail.com (M.K.); higo@nms.ac.jp (S.H.); yujiro@nms.ac.jp (Y.H.); hozawa@nms.ac.jp (H.O.); 2 Course of Oriental Medical Care Teacher Training, Tokyo School of Medical Treatment and Welfare, 1-11-11, Hatchobori, Chuo-ku, Tokyo 104-0032, Japan

**Keywords:** alternative promoter usage, alternative splicing, antibody validation, ESR2

## Abstract

Several lines of controversial evidence concerning estrogen receptor β (ERβ) remain to be solved because of the unavailability of specific antibodies against ERβ. The recent validation analysis identified a monoclonal antibody (PPZ0506) with sufficient specificity against human ERβ. However, the specificity and cross-reactivity of PPZ0506 antibody against ERβ proteins from laboratory animals have not been confirmed. In the present study, we aimed to validate the applicability of PPZ0506 to rodent studies. The antibody exhibited specific cross-reactivity against mouse and rat ERβ proteins in immunoblot and immunocytochemical experiments using transfected cells. In immunohistochemistry for rat tissue sections, PPZ0506 showed immunoreactive signals in the ovary, prostate, and brain. These immunohistochemical profiles of rat ERβ proteins in rat tissues accord well with its mRNA expression patterns. Although the antibody was reported to show the moderate signals in human testis, no immunoreactive signals were observed in rat testis. Subsequent RT-PCR analysis revealed that this species difference in ERβ expression resulted from different expression profiles related to the alternative promoter usage between humans and rats. In conclusion, we confirmed applicability of PPZ0506 for rodent ERβ studies, and our results provide a fundamental basis for further examination of ERβ functions.

## 1. Introduction

Ovarian steroid hormones, estrogens, play an important role in signal transduction pathways across various organs in females and males [1]. Estrogens signal via activation of nuclear estrogen receptors (ERs). Two subtypes of ERs, ERα and ERβ (also termed NR3A1 and NR3A2 [2], and with the symbols ESR1 and ESR2, respectively), belong to the nuclear steroid receptor superfamily and function as ligand-inducible transcription regulators. They are widely expressed in a wide variety of organs and exhibit distinct expression patterns.

Expressions of ER genes are regulated by multiple promoter systems [3]. Alternative promoter usage of ERα genes has been analyzed [4], and our comparative studies of these systems reported conserved and species-specific genomic structures and expression profiles among humans, mice, and rats [5,6,7,8,9]. Transcription of ERβ genes is initiated by activation of multiple promoters. Human and rat ERβ genes were reported to possess three promoters (E1, 0N, and 0K in humans, and E1/P2, 0N/P1, and 0H in rats) [10,11,12,13,14,15]. However, few studies have reported on species differences in patterns of alternative ERβ gene promoter usage.

ERβ cDNA was first discovered in the rat prostate [16], and the initial studies of Kuiper et al. [16,17] documented its high expression in rat prostate and ovary and low expression in rat testis. In contrast, human ERβ cDNA was first cloned from the testis [18], and its abundant expression in the human testis was reported [18,19]. Although species differences in testicular ERβ expression probably exist between humans and rats, this difference has not been reported because several immunohistochemical studies documented intense immunoreactive signals against ERβ proteins in rat testis. However, the testicular localization of rat ERβ proteins are controversial, ranging from limited cell types to most cell types in the testis [20,21,22,23]. This disparity between the expression levels of ERβ mRNAs and proteins and the inconsistency in ERβ protein localization are thought to result from the lack of well validated antibodies against ERβ proteins.

Although several efforts have been made to validate anti-ERβ antibodies [24,25,26], anti-ERβ antibodies with specific reactivity and wide application are not commercially available. This paucity has resulted in multiple lines of disconcordant evidence generated by the use of inadequately validated antibodies, which has distorted ERβ research. Recently, Andersson et al. [27] evaluated the immunoreactivities of thirteen anti-ERβ antibodies and reported that only one commercial anti-human ERβ antibody (PPZ0506) reacted specifically against human ERβ proteins and was applicable to immunohistochemical experiments using human cells and tissues. This antibody was expected to be a helpful tool to solve contradictory problems concerning ERβ, and actually used for specific detection of human ERβ proteins in normal and malignant breast tissues [28]. However, the specificity and cross-reactivity of PPZ0506 antibody against ERβ proteins from laboratory animals have not been confirmed, and its application has been limited to human-derived samples. Andersson et al. [27] demonstrated the abundant expression of ERβ mRNAs and moderate immunoreactive signals against ERβ proteins in human testis. However, no concordant data on testicular expression levels of ERβ mRNAs and proteins and their localization have been reported in the rodents. Therefore, assessing the specificity and cross-reactivity of PPZ0506 antibody against rodent ERβ proteins is required to solve the disconcordant expression profiles of rodent ERβ.

To solve several contradictory problems in ERβ research, we focused on the discrepant expression profiles of rat ERβ mRNAs and proteins. In the present study, we evaluated the specific immunoreactivity of PPZ0506 antibody against rodent ERβ proteins and its ability to examine the expression of rat ERβ proteins in rat tissues using immunohistochemistry. Moreover, to determine the species–specific transcriptional regulation of ERβ genes, profiles of their alternative promoter usage were comparatively assessed between humans and rats.

## 2. Results

### 2.1. Specificity and Cross-Reactivity of PPZ0506 Antibody Against Human, Mouse, and Rat ERβ Proteins

#### 2.1.1. Construction of Expression Vectors Encoding Human, Mouse, and Rat ERβ

Kuiper et al. [16] first cloned ERβ cDNA from rat prostate, and their registered sequence (U57439) was used as the rat ERβ reference (RefSeq, NM_012754). However, subsequent cloning studies of other groups reported nucleotide sequences (O’Brien et al. [15], AJ002602, AB190769, and AB190770) encoding rat ERβ proteins with a 64 additional N-terminal amino acid related to one extra base alteration in the 5′-region. Since the PPZ0506 antibody was developed using an N-terminal peptide sequence of human ERβ (2–88 amino acids) as an antigen, this discrepancy in rat ERβ information has hindered the specificity and cross-reactivity of the antibody against rat ERβ proteins from being assessed. To construct expression vectors encoding full-length ERβ proteins, the 5′-region of rat ERβ cDNA was cloned and analyzed by DNA sequencing (Supplementary Figure S1a). The analysis confirmed the extra base insertion in the 5′-region of the rat ERβ gene. We cloned human, mouse, and rat full-length ERβ (ERβ1), and mouse and rat ERβ splice variant (ERβ_ins_) sequences [29,30,31,32] and introduced them into pCMV-Tag 2B vectors with FLAG tag (DYKDDDDK) sequences in-frame (Supplementary Figure S1b,c). Successful construction of the expression vectors and functional expression of the encoded proteins were confirmed in transfected HEK293 cells using luciferase reporter assays (Supplementary Figure S2). ERα and ERβ1 constructs exhibited ligand-inducible transactivation of an estrogen response element (ERE)-driven promoter (Supplementary Figure S2a), whereas ER constructs did not modulate the transactivation of an ERE-less minimum promoter (Supplementary Figure S2b). Functions of ERβ_ins_ constructs were analyzed using competitive assays (Supplementary Figure S2c–f). ERβ_ins_ repressed full-length ERα (ERα66)- and ERβ1-mediated transactivation.

#### 2.1.2. Immunoblot Analyses of Human, Mouse, and Rat ERβ in Transfected Cells

**Figure 1:**
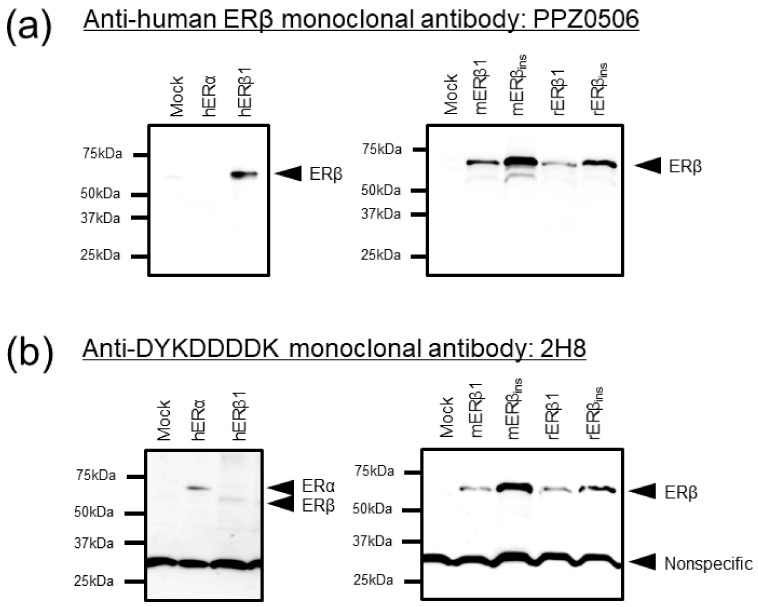
Confirmation of specific immunoreactivity of PPZ0506 antibody against human, mouse, and rat estrogen receptor β (ERβ) proteins in immunoblotting analysis. (**a**) Immunoblot detection of human, mouse, and rat ERβ proteins in transfected HEK293 cells using anti-human ERβ monoclonal antibody (PPZ0506). (**b**) Immunoblot detection of FLAG (DYKDDDDK)-tagged ERα and ERβ proteins in transfected HEK293 cells using anti-DYKDDDDK monoclonal antibody (2H8). “h”, “m”, and “r” indicate human, mouse, and rat, respectively. Mock-transfected cells (mock) were used as negative controls. An equal amount of protein lysate was loaded in each lane (0.5 μg/lane). The representative images in panels (**a**) and (**b**) were obtained in parallel using the same samples. Similar results were obtained in three separate experiments (*n* = 3).

Immunoblot analyses were performed to confirm the specificity and cross-reactivity of PPZ0506 antibody against human, mouse, and rat ERβ proteins. Expression vectors encoding FLAG-tagged ERα and ERβ were introduced into HEK293 cells, and immunoreactivity against ERβ proteins was evaluated using lysates of the transfected cells (Figure 1). PPZ0506 antibody reacted with human, mouse, and rat ERβ proteins, whereas no immunoreactive signals were observed in mock- or ERα-transfected cell lysates (Figure 1a). Successful induction of the constructs was confirmed by immunoblot analyses using an anti-FLAG antibody (2H8; Figure 1b).

#### 2.1.3. Immunocytochemical Analyses of Human, Mouse, and Rat ERβ in Transfected Cells

**Figure 2:**
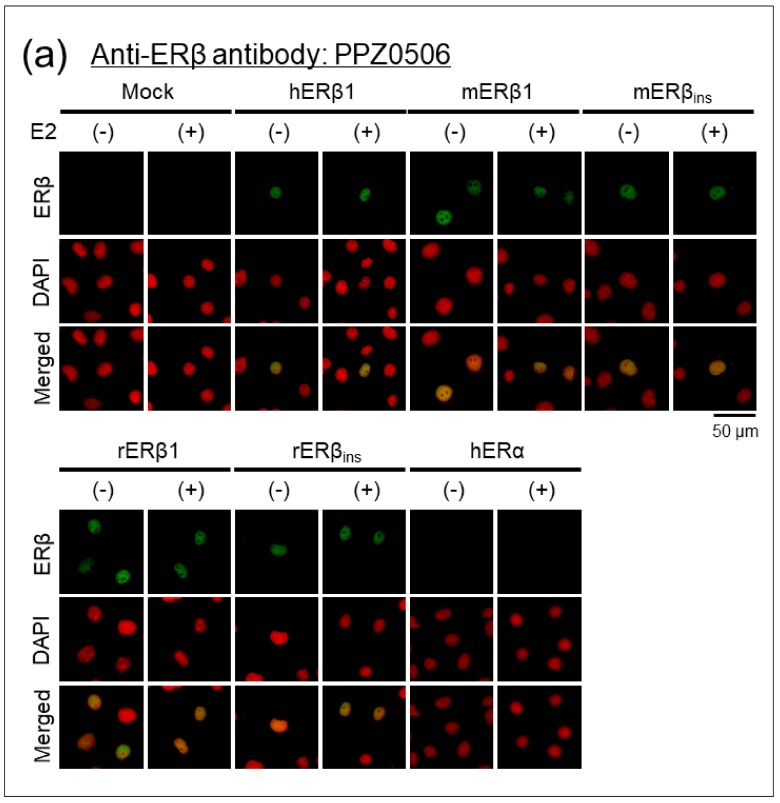

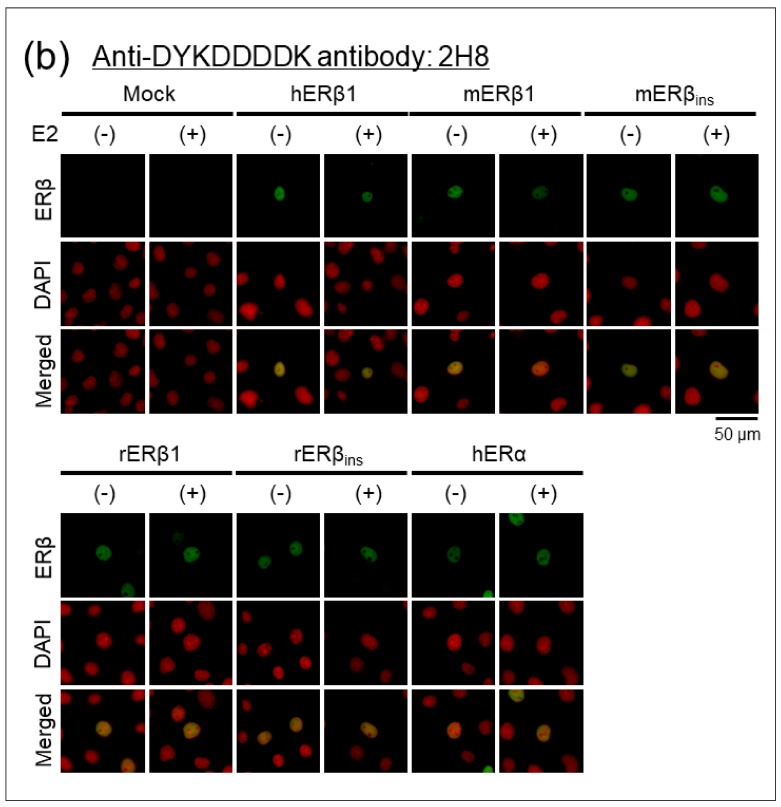
Confirmation of specific immunoreactivity of PPZ0506 antibody against human, mouse, and rat ERβ proteins in immunocytochemical analyses. (**a**) Immunocytochemical detection of human, mouse, and rat ERβ proteins in transfected COS-7 cells using anti-human ERβ monoclonal antibody (PPZ0506). (**b**) Immunocytochemical detection of FLAG-tagged ERα and ERβ proteins in transfected COS-7 cells using anti-DYKDDDDK monoclonal antibody (2H8). Transfected cells were treated with 10 nM E_2_ (+) or 0.1% EtOH (–). “h”, “m”, and “r” indicate human, mouse, and rat, respectively. Mock-transfected cells (mock) were used as negative controls. Alexa Fluor 488 and 4′,6-diamino-2-phenylindole (DAPI) images were pseudocolored in green and red, respectively. Scale bar: 50 μm. Similar results were obtained in three separate experiments (*n* = 3).

Specificity and cross-reactivity of PPZ0506 antibody were evaluated in immunocytochemical experiments using the transfected cells. The COS-7 cell line was used as host cells because COS-7 cells are more adhesive to culture dishes than HEK293 cells. COS-7 cells were transfected with the expression constructs and treated with 10 nM 17β-estradiol (E_2_) or 0.1% ethanol (EtOH) as a vehicle. Immunoreactive signals of PPZ0506 antibody were observed in the nuclei of cells transfected with human, mouse, and rat ERβ constructs, whereas no immunofluorescent signals were found in mock- or ERα-transfected cells (Figure 2a). Appropriate expression of the FLAG-tagged constructs was confirmed using 2H8 antibody (Figure 2b). Subcellular localization of ER proteins was not altered in the presence or absence of E_2_.

### 2.2. Immunohistochemical Analyses of ERβ Proteins in Rat Organs

#### 2.2.1. Expression of ERβ Proteins in Rat Ovary

**Figure 3:**
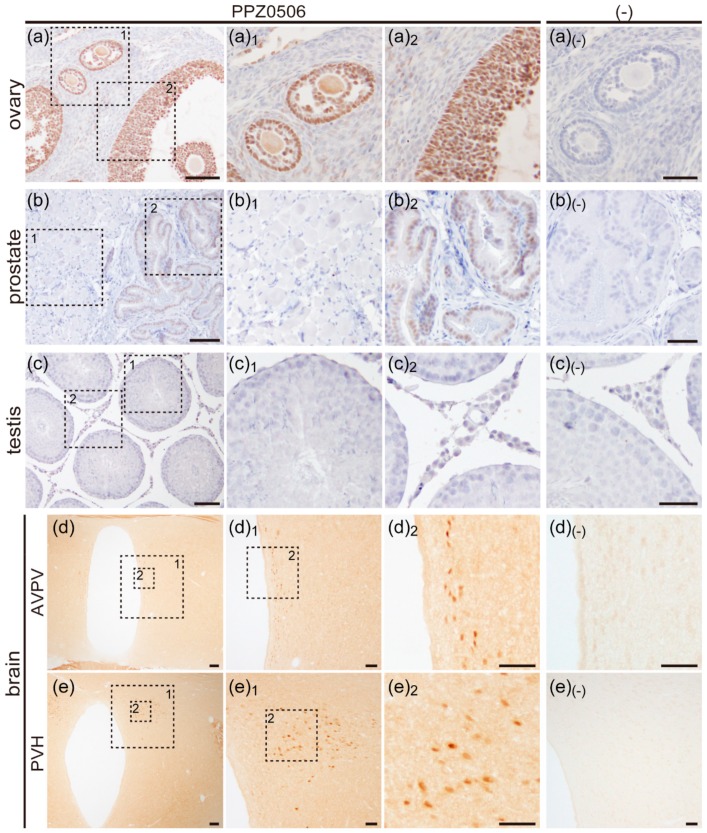

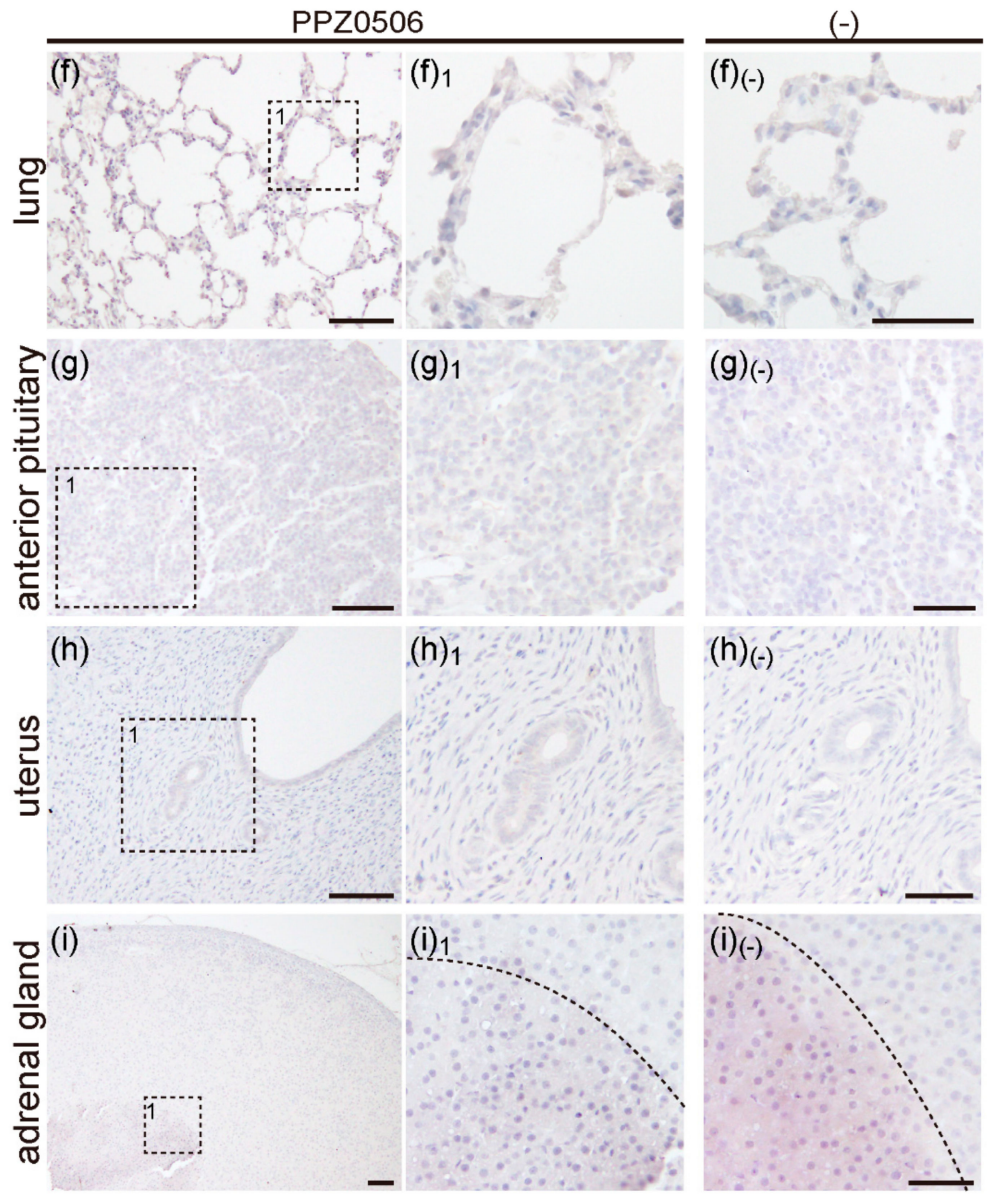
Immunohistochemical analysis of rat ERβ expression in rat tissues. Immunohistochemical signals against rat ERβ proteins were evaluated in the ovary (**a**), prostate (**b**), testis (**c**), AVPV (**d**), PVH (**e**), lung (**f**), anterior pituitary (**g**), uterus (**h**), and adrenal gland (**i**). Left panels (**a**–**i**), low magnification; middle panels (**a_1_**–**i_1_**, **a_2_**–**e_2_**), magnified images of the framed areas in the left panels; right panels (**a_(-)_**–**i_(-)_**), immunostaining without PPZ0506 antibody; the brain sections are thicker (16 μm) than the other sections (5 μm) and not counterstained with hematoxylin. The dotted lines in panels (**i_1_**) and (**i_(-)_**) indicate boundaries between the adrenal cortex and medulla. Scale bars: 100 μm in left panels; 50 μm in middle and right panels. Similar results were obtained in three separate experiments (*n* = 3).

Rat paraffin-embedded ovary sections were used in immunohistochemical experiments. In our preliminary experiments, rat ovaries at the estrus stage exhibited weaker immunoreactive signals against ERβ proteins than those at diestrus and proestrus stages. Thus, sections of diestrus ovaries were used for immunohistochemical analyses (Figure 3a). Dense immunoreactive signals against ERβ were detected in granulosa cells. Weakly stained stromal cells and faintly stained theca cells were dispersedly observed. The signals were predominantly localized in their nuclei. Experiments without the primary anti-ERβ antibody displayed no immunoreactive signals (Figure 3a_(-)_).

#### 2.2.2. Expression of ERβ Proteins in Rat Prostate

Rat prostate sections were immunostained using PPZ0506 antibody. Immunoreactive signals against ERβ were detected only in the nuclei of epithelial cells (Figure 3b). Experiments without the primary antibody exhibited no immunoreactivity (Figure 3b_(-)_).

#### 2.2.3. Expression of ERβ Proteins in Rat Testis

Rat testis sections were prepared and immunostained using PPZ0506 antibody (Figure 3c). Immunoreactive signals against ERβ proteins were not detected in rat testis.

#### 2.2.4. Expression of ERβ Proteins in Rat Brain

Female rat brains at the diestrus stage were fixed by perfusion. Frozen brain sections containing the anteroventral paraventricular nucleus (AVPV) and the paraventricular nucleus of hypothalamus (PVH) were prepared and immunostained using PPZ0506 antibody (Figure 3d,e). Immunoreactive cell populations were observed in the AVPV and PVH. Experiments without the primary antibody exhibited no immunoreactivity (Figure 3d_(-)_,e_(-)_).

#### 2.2.5. Expression of ERβ Proteins in Rat Lung, Anterior Pituitary, Uterus and, Adrenal Gland

Paraffin-embedded sections of rat lungs, anterior pituitary, uteri, and adrenal glands were prepared and immunostained using PPZ0506 antibody (Figure 3f–i). No immunoreactive signals against ERβ proteins were detected in the sections.

### 2.3. Alternative Promoter Usage Profiles of Human and Rat ERβ Genes

#### 2.3.1. Genomic Organization of Human and Rat ERβ Genes

The alternative promoter systems of ER genes contain several promoter-specific leader exons and untranslated internal exons in the 5′-regions, and alternative promoter usage and alternative splicing of the untranslated exons yield multiple ER variants with distinct 5′-ends. The human ERβ gene possesses three promoters. The presence of untranslated internal exons and 5′-untranslated region (5′-UTR) variants inserted near the exons is controversial: Moore et al. [19] and Lee et al. [10] reported the alternative inclusion of multiple untranslated internal exons (0X1–0X8) between exons 0K and 1, whereas Hirata et al. [14] and Smith et al. [11,12] did not report their presence. The rat ERβ gene was reported to contain three promoters (E1/P2, 0N/P1, and 0H) [13,15], but no untranslated internal exons have been documented.

**Figure 4:**
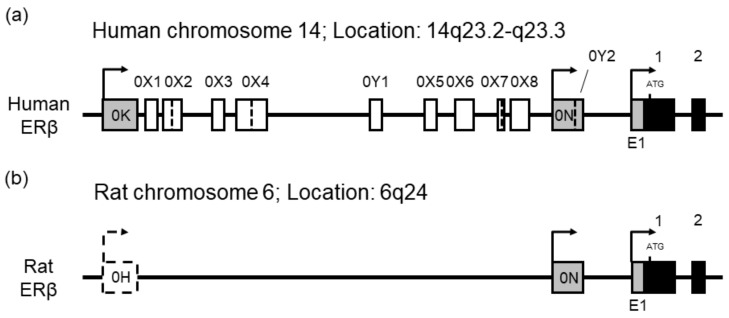
Genomic organization of the 5′-regions of human and rat ERβ genes. Structure of the 5′-regions of the (**a**) human and (**b**) rat ERβ gene, represented schematically. Human and rat ERβ genes are located at 14q23.2-23.3 on human chromosome 14 and at 6q24 on rat chromosome 6. Black, gray, and white boxes indicate coding exons, untranslated leader exons, and untranslated internal exons, respectively. Exon 0Y2 localizes to the 3′-end of human exon 0N. Exons 0X2, 0X4, and 0X7 have two alternative splice donor sites, and human exon 0N contains a splice acceptor site of exon 0Y2. Dotted lines in boxes and bent arrows represent alternative splice sites and transcriptional start sites, respectively. Nucleotide sequences of the leader and internal exons are shown in detail in Supplementary Figure S5. Human leader exons E1 and 0N are orthologous to rat leader exons E1/P2 and 0N/P1, respectively. Human leader exon 0K is not homologous to rat leader exon 0H. The image is not to scale.

To isolate human and rat ERβ 5′-UTR variants and determine the genomic organization of the 5′-regions of human and rat ERβ genes, the rapid amplification of cDNA 5′-end (5′-RACE) cloning was performed. Human testis, and rat ovary and prostate total RNAs were used to generate 5′-RACE cDNAs, and human and rat ERβ 5′-UTR variants were amplified by nested PCRs. From human testis 5′-RACE cDNA, we cloned E1-1, 0N-1, and 0K-1 variants and further identified several 0K isoforms containing internal sequences. RT-PCR cloning was carried out with total RNA from the testis (Supplementary Figure S3). Forward PCR primers were designed for respective leader exons to amplify promoter-specific isoforms. The open reading frame (ORF) region between exons 1 and 4 was amplified to evaluate overall ERβ expression. We obtained a single amplicon of the E1 and 0N isoforms, whereas several bands were observed for amplification of the 0K isoforms. Furthermore, when the primer pairs used in previous reports by Hirata et al. [14] and Smith et al. [11,12] were applied to the PCR amplification of 0K isoforms (Supplemental Figure S4) we found band patterns of 0K isoforms similar to our initial result. DNA sequencing revealed that the multiple amplicons were generated by the insertion of nucleotide sequences between exons 0K and 1. The composition of the ERβ clones identified in 5′-RACE and RT-PCR experiments are shown in Supplementary Table S1. The subscripts, “L” and “S” indicate long and short forms generated by alternative usage of splice donor sites, respectively. We confirmed the alternative inclusion of exons 0X1–0X8 between exons 0K and 1 and identified two novel untranslated internal exons and their alternative insertion. We named the novel internal exons “0Y” and numbered them according to their 5′ to 3′ order in the gene (exons 0Y1 and 0Y2). The genomic organization of the 5′-region of the human ERβ gene is schematically represented in Figure 4. Nucleotide sequences of the 5′-UTR exons are shown in detail in Supplemental Figure S5. Exon 0Y2 is located in the 3′-end of leader exon 0N. From rat prostate and ovary 5′-RACE cDNAs, we cloned E1/P2-1 and 0N/P1-1 variants, but did not observe amplification of rat 0H isoforms. Rat 0H isoforms were not detected using RT-PCR.

#### 2.3.2. Alternative Promoter Usage of Human ERβ Gene

**Figure 5:**
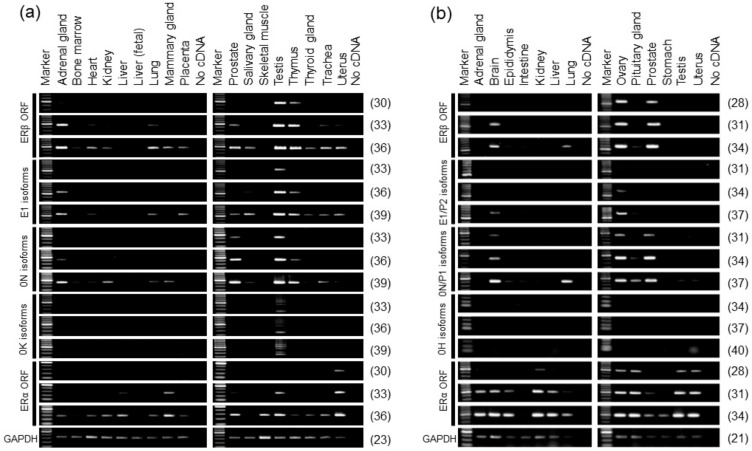
Profiles of the alternative promoter usage of human and rat ERβ genes. (**a**) Expression patterns of human promoter-specific ERβ variants. (**b**) Expression patterns of rat promoter-specific ERβ variants. Total RNAs isolated from multiple organs were subjected to RT-PCR. The distribution and splice patterns of human E1, 0N, and 0K isoforms, and rat E1/P2 and 0N/P1 isoforms were analyzed. To assess the overall expression of the human and rat ERβ genes, the open reading frame (ORF) regions were amplified (ERβ ORF). As a comparison, the expression of the human and rat ERα genes was evaluated by amplifying the coding regions between human exons 2 and 3, and rat exons 6 and 8, respectively (ERα ORF). *GAPDH* was used as an internal control. From the upper to lower panels, the number of PCR cycles increased in increments of three cycles (indicated to the right of the respective panels). Human cDNAs require two more PCR cycles than rat cDNAs to detect target molecules due to the quality of their total RNAs.

Profiles of alternative promoter usage of the human ERβ gene were analyzed in multiple organs using RT-PCR (Figure 5). The overall expression of human ERβ mRNA was estimated by amplifying the coding region between exons 1 and 4. ERβ mRNAs were widely detected in the human organs except the liver and skeletal muscle. The testis exhibited the highest expression levels of ERβ mRNAs in the human organs analyzed. Human ERβ mRNAs were highly expressed in the adrenal gland and thymus, and moderately expressed in the lung, prostate, trachea, and uterus. Human E1 isoforms were expressed highly in the adrenal gland, testis, and thymus, and they were moderately expressed in the salivary gland. Human 0N isoforms were highly expressed in the adrenal gland, prostate, testis, and thymus, and they were detectable in the other ERβ-positive organs. The 0K isoforms were expressed predominantly in the testis. As a comparison, the expression profiles of human ERα mRNA were assessed by amplifying the coding region between exons 2 and 3.

#### 2.3.3. Alternative Promoter Usage of Rat ERβ Gene

Profiles of the alternative promoter usage of rat ERβ gene were evaluated using RT-PCR (Figure 5). The overall expression of rat ERβ mRNA was estimated by amplifying the coding region between exons 7 and 8. Rat ERβ mRNAs were strongly expressed in the ovary and prostate, moderately expressed in the brain, and weakly in the lung. Faint expression of ERβ mRNAs was observed in the pituitary gland. Rat E1/P2 isoforms were strongly expressed in the ovary, and they were weakly expressed in the brain. Rat 0N/P1 isoforms were expressed highly in the brain, lung, ovary, and prostate, and moderately expressed in the pituitary gland. Rat 0H isoforms were not detected. As a comparison, the expression patterns of rat ERα mRNA were evaluated by amplifying the coding region between exons 6 and 8.

## 3. Discussion

In the present study, we demonstrated that the anti-human ERβ monoclonal antibody, PPZ0506, reacted specifically with human, mouse, and rat ERβ proteins in immunoblot and immunocytochemical experiments using transfected cells. Furthermore, we detected immunoreactive signals against rat ERβ proteins in the ovary, prostate, and brain. Although Andersson et al. [27] reported moderate immunohistochemical signals against ERβ proteins in human testis using the antibody, reactivity against ERβ protein was scarce in rat testis. This discrepancy of testicular immunoreactive profiles between humans and rats resulted from the species–specific regulation of the ERβ genes.

Since specific and well validated antibodies against ERβ proteins were not commercially available, several lines of controversial evidence concerning ERβ have remained to be solved. The recent discovery of a well validated monoclonal antibody (PPZ0506) against human ERβ proteins has prompted attempts to solve the conflicting problems in ERβ research. However, the specificity and cross-reactivity of PPZ0506 against ERβ proteins from laboratory animals was not determined. This was related to uncertain information regarding the N-terminal sequences of rodent ERβ proteins [29]. The PPZ0506 antibody was developed using an N-terminal peptide of human ERβ (2–88 amino acids) as an antigen. The rat ERβ reference (RefSeq, NM_012754) and its original sequence (U57439) contain a one nucleotide deletion in their 5′-region and therefore encode N-terminally truncated ERβ proteins that lack the full epitopic region for the PPZ0506 antibody. We cloned rat ERβ sequences and confirmed that ERβ proteins with a longer N-terminal and full epitope are potentially encoded by rat ERβ cDNAs. Specific immunoreaction of the PPZ0506 antibody against mouse and rat ERβ proteins were determined in transfected cells using expression constructs encoding full-length ERβ proteins. The PPZ0506 antibody was applicable to immunoblot and immunocytochemical experiments using transfected cells. Although the mitochondrial and plasma membrane localization of ERβ proteins were reported [33,34], the extranuclear localization of ERβ proteins were not observed in our immunocytochemical experiments using transfected COS-7 cells.

In our immunohistochemical experiments, immunoreactive signals against rat ERβ proteins were detected only in the ovary, prostate, and brain, but not observed in the adrenal gland, lung, anterior pituitary, testis, and uterus. Rat ERβ proteins were abundantly expressed in granulosa cells of the ovary, epithelial cells of the prostate, and cell populations in the AVPV and PVH of the brain. Their signals localized mainly in their nuclei. Pioneering studies of Saunders et al. [22,23] documented rat ERβ expression in a wide range of tissues and cell types. RT-PCR analysis revealed narrow expression profiles of rat ERβ mRNAs: They are highly expressed in the ovary and prostate, moderately in the brain, and weakly in the lung and pituitary gland. The other organs tested scarcely express rat ERβ transcripts. These expression profiles of rat ERβ gene accord well with the immunoreactive profiles of rat ERβ proteins using PPZ0506 antibody. The organs in which rat ERβ mRNAs are detectable below 31 PCR cycles are immunoreactive. Comparative analysis of human and rat ERβ gene expression revealed their species–specific regulation. Human ERβ mRNAs are widely distributed in multiple organs, and highly expressed in the adrenal gland and testis. Unlike human testicular ERβ, rat ERβ mRNAs were faintly expressed in the testis, which was reflected by scarce immunoreactive signals against rat testicular ERβ proteins in the present study. These species differences in ERβ expression between humans and rats was attributed to distinct profiles of alternative promoter usage. Three distinct promoters were vigorously activated in human testis, whereas two different promoters were not utilized in rat testis. Furthermore, human E1 and 0N promoters were widely activated.

Alternative promoter usage and alternative splicing of ERβ genes generate multiple ERβ variants with unique 5′-UTRs, which contribute to post-transcriptional regulation of the ERβ transcripts [10,11,12]. Human ERβ isoforms were predominantly expressed in human testis, and the 5′-UTRs of human 0K isoforms were reported to repress translational efficiency of their transcripts [10]. Andersson et al. [27] documented the abundant expression of ERβ mRNAs and moderate immunoreactive signals of ERβ proteins in human testis. This difference between ERβ mRNA and protein levels in human testis is partly related to the post-transcriptional repression of human 0K isoforms.

The alternative inclusion of 5′-untranslated internal exons contributes to the remarkable mRNA diversity of the ERα genes [6,7,8,9]. However, the existence of 5′-UTR ERβ variants by the insertion of untranslated exons has been debated. The present study solved this conflict and supported the presence of multiple 0K isoforms. Furthermore, two novel untranslated internal exons (0Y1 and 0Y2) were identified in the 5′-region of the human ERβ gene. Our successful detection of the variants is related to the use of human testis total RNA as a target that contains abundant 0K isoform transcripts. Although Hirata et al. [14] and Smith et al. [11,12] did not observe the presence of multiple 0K isoforms, RT-PCR with primer pairs used in their studies amplified multiple 0K isoforms from human testis total RNA.

In conclusion, the rat ERβ gene potentially encodes a protein with a longer N-terminal than originally thought, and the monoclonal anti-human ERβ antibody, PPZ0506, exhibited specific immunoreaction against human, mouse, and rat full-length ERβ proteins and was applicable for immunoblot and immunocytochemical experiments using transfected cells. Immunohistochemical profiles of rat ERβ proteins using PPZ0506 accord well with expression patterns of rat ERβ gene. The disconcordant immunoreactive signals against human and rat ERβ proteins reflects the species-distinct regulation of ERβ genes. In the present study, some conflicting problems in ERβ research were solved; however, several lines of controversial evidence concerning ERβ remain to be solved. We expect that our results will prompt attempts to understand the fundamental features of ERβ further.

## 4. Materials and Methods

### 4.1. Animals

All experiments using animals in this study were approved by the Nippon Medical School Animal Care and Use Committee and conducted according to the institutional guidelines. Eight-to-twelve-week-old Wistar rats were purchased from Tokyo Laboratory Animals Science (Tokyo, Japan). The animals were fed ad libitum and housed in a temperature-controlled room (22–24 °C) under a 14 h light/10 h dark cycle. For extraction of total RNAs and preparation of paraffin sections, the animals were euthanized under deep anesthesia with sodium pentobarbital (150 mg/kg, i.p.). Their organs were removed and rinsed in phosphate-buffered saline (PBS). For total RNA extraction, the removed organs were frozen in liquid nitrogen until use. For immunohistochemical experiments, the organs were fixed in fixative solution (4% (*w*/*v*) paraformaldehyde/0.1 M phosphate buffer (PB, pH 7.4)) for 24 h at 4 °C. To prepare paraffin sections, the fixed organs were dehydrated through a graded ethanol series and embedded in paraffin. For preparation of frozen brain sections, the animals were transcardially perfused with 50 mL of saline followed by 200 mL of fixative solution under deep anesthesia with a mixture of sodium pentobarbital (50 mg/kg, i.p.) and medetomidine hydrochloride (0.5 mg/kg, i.p.). Brains were post-fixed in the same fixative for 24 h at 4 °C, and cryoprotected in 0.1 M PB containing 20% (*w*/*v*) sucrose and 0.9% (*w*/*v*) NaCl.

### 4.2. Total RNA Extraction, 5′-RACE, and RT-PCR

Total RNAs were extracted from rat organs using RNAiso Plus (Takara Bio, Shiga, Japan) according to the manufacturer’s protocols. The extracted total RNAs were treated with Turbo DNase (Thermo Fisher Scientific, Waltham, MA, USA) and re-purified. Human total RNAs were purchased from Takara Bio and Thermo Fisher Scientific. The information of human total RNAs sources was described previously [35]. Human and rat total RNAs were reverse-transcribed using oligo(dT)_15_ primers. Reaction mixtures (25 μL) contained 5 μg of total RNA, 1× RT buffer, 1 mM dNTP mixture, 1 μg of oligo(dT)_15_ primers, 20 U RNase inhibitor (Takara Bio), and 100 U RTase (ReverTra Ace; Toyobo, Osaka, Japan). The reaction was carried out at 42 °C for 60 min and stopped by heating at 75 °C for 15 min. 5′-RACE cDNA was synthesized from human testis total RNA, and rat prostate and ovary total RNAs were synthesized using a SMARTer RACE cDNA Amplification Kit (Takara Bio) according to the manufacturer’s instructions. For vector construction, coding sequences of human, mouse, and rat ERβ1, and mouse and rat ERβ_ins_ (also known as mouse and rat ERβ2 [30,31,32]) were amplified using KOD-*Plus*-Neo polymerase (Toyobo). 5′-RACE products were amplified by two rounds of PCR with 5′-RACE universal primers and ERβ-specific primers using KOD-*Plus*-Neo polymerase. For expression analyses, Blend Taq polymerase (Toyobo) was used. The PCR mixture (25 μL) contained cDNA corresponding to 25 ng of total RNA, 1× PCR buffer, 0.2 mM dNTP mixture, 0.2 μM each forward and reverse primers, 0.63 U Blend Taq polymerase. PCR was performed in three steps: A PCR cycle reaction of 95 °C for 30 s, 60 °C for 20 s, and 72 °C for 30 s, with an initial denaturing step of 95 °C and 5 min and a final elongation step of 72 °C for 5 min. Oligonucleotide primers used for 5′-RACE and RT-PCR were purchased from Nihon Gene Research Laboratories (Sendai, Japan), and listed in Supplementary Table S2. To avoid amplifying any contaminating genomic DNA, the PCR primer pairs were designed from different exons. PCR products were cloned into pGEM-T-Easy vectors (Promega Corporation, Madison, WI, USA) and DNA-sequenced.

### 4.3. Vector Construction

The human ERα coding sequence was amplified from pcDNA3.1-human ERα66 [5] using KOD -*Plus*- Neo polymerase with restriction site-adapted primers, cloned into a pCMV-Tag 2B vector (Agilent Technologies, Santa Clara, CA, USA), and fused in-frame with a FLAG epitope tag sequence (pFLAG-hERα66). Human, mouse, and rat ERβ coding sequences were amplified from the pGEM-T-Easy vectors prepared above and cloned into pCMV-Tag 2B vectors (pFLAG-hERβ1, pFLAG-mERβ1, pFLAG-mERβ_ins_, pFLAG-rERβ1, and pFLAG-rERβ_ins_). The cloned fragments were confirmed by DNA sequencing analyses.

### 4.4. Cell Culture and Transfection

Authenticated cell lines, COS-7 (Cell #. JCRB9127) and HEK293 (JCRB9068), were obtained from the Japanese Collection of Research Bioresources Cell Bank (Osaka, Japan). The cells were cultured in Dulbecco’s modified Eagle medium (DMEM) supplemented with 10% fetal bovine serum (FBS; Equitech-Bio, Kerrville, TX, USA) and 1% penicillin/streptomycin solution (Fujifilm Wako Pure Chemical Corporation, Osaka, Japan). The cells were maintained according to our previous studies [35,36]. For transfection, cells were cultured on appropriate culture plates or dishes, with lipofection of plasmid vectors using TransFast Transfection Reagents (Promega) according to the manufacturer’s protocols.

### 4.5. Immunoblotting

HEK293 cells were seeded on 6-well culture plates (Techno Plastic Products, Trasadingen, Switzerland) and transfected with 1 μg of vector constructs. Forty-eight hours after transfection, the cells were washed twice with PBS and harvested in RIPA buffer containing 1× Protease Inhibitor Cocktail Set (Fujifilm Wako). Cell lysates were mixed with an SDS sample buffer. Equal amounts (0.5 μg) of protein samples were loaded in the respective lanes and electrophoresed on gradient polyacrylamide gels (ATTO Corporation, Tokyo, Japan). Electrophoresed proteins were transferred to polyvinylidene fluoride membranes and reacted with anti-DYKDDDDK (Clone # 2H8; dilution rate, 1:1000; TransGenic, Fukuoka, Japan) or anti-ERβ (PPZ0506; 1:2000; Perseus Proteomics, Tokyo, Japan) monoclonal antibodies in 0.3% skim milk/0.1% Tween-20/Tris-buffered saline (TBS; 25 mM Tris-HCl (pH 7.4), 0.15 NaCl) for 16 h. After washing three times in 0.1% Tween-20/TBS, the membranes were incubated with anti-mouse IgG, horseradish peroxidase-linked antibody (Lot # 31; 1:2500; Cell Signaling Technology, Danvers, MA, USA) in 0.3% skim milk/0.1% Tween-20/TBS for 1 h. Immunoluminescent signals were detected with ImmunoStar Zeta substrates (Fujifilm Wako) using an ImageQuant LAS 4000 System (GE Healthcare, Chicago, IL, USA).

### 4.6. Immunocytochemistry

COS-7 cells were seeded on collagen-coated glass bottom dishes (Matsunami Glass, Osaka Japan) and transfected with 500 ng of respective vector constructs. Twenty-four hours after transfection, the cells were treated with 10 nM E_2_ (Merck, Kenilworth, NJ, USA) or 0.1% EtOH as a vehicle in DMEM with 2.5% charcoal-stripped FBS (Equitech-Bio) and 1% penicillin/streptomycin solution for another 24 h. Transfected and mock-transfected cells were washed three times with PBS and fixed in 10% formalin/PBS for 1 h. The fixed cells were permeabilized in 0.1% Triton X-100/PBS for 5 min and treated with 2H8 or PPZ0506 antibodies in 0.3% bovine serum albumin (BSA)/0.1% Tween-20/PBS for 16 h. After washing three times in 0.1% Tween-20/PBS, the cells were incubated with an Alexa Fluor 488-labeled goat anti-mouse IgG secondary antibody (Lot # 1975516; 1:250; Thermo Fisher Scientific) in 0.3% BSA/0.1% Tween-20/PBS for 1 h. Nuclei of cells were counterstained with DAPI (Fujifilm Wako). Immunofluorescent images were captured using a BZ-8000 Fluorescence Microscope System (Keyence, Osaka, Japan). Alexa Fluor 488 and DAPI images were pseudocolored in green and red, respectively.

### 4.7. Immunohistochemistry

Paraffin-embedded organs were sliced into 5 μm tissue sections on a Leica RM2235 Rotary Microtome (Leica Microsystems, Wetzlar, Germany), and transferred to frontier coated slides (Matsunami Glass). The paraffin sections were then deparaffinized and rehydrated. Frozen brains were coronally sliced into sections at 16 μm thickness using a cryostat (Leica 3050, Heidelberg, Germany), and collected into PBS. The sections containing the AVPV (approximately AP −0.3 to −0.5 mm from Bregma), and the PVH (approximately AP −1.5 to −1.9 mm from Bregma) were mounted on the slide glasses. Immunohistochemistry was performed using a streptavidin-horseradish peroxidase-based kit (Histofine SAB-PO kit; Nichirei Corporation, Tokyo, Japan). Sections were treated with 0.3% (*v*/*v*) H_2_O_2_ in methanol to quench endogenous peroxidase activity, and then washed three times in PBS. Antigen retrieval was performed by autoclaving sections for 10 min at 121 °C in a buffer containing 10 mM citric acid and 0.05% Tween-20 (pH 6.0). After blocking in 5% (*v*/*v*) normal rabbit serum, sections were immersed in PPZ0506 antibody at dilution of 1:400 for the brain and 1:500 for the other organs in 0.1% Triton X-100/PBS and incubated for 16 h at 4 °C. Staining was developed using 3,3′-diaminobenzidine tetrahydrochloride (Merck) with 0.01% hydrogen peroxide. After washing three times in TBS, the sections except the brain ones were counterstained with Lillie Mayer’s hematoxylin. The sections were dehydrated through a graded ethanol series, cleared in xylene, and mounted with Permount Mounting Medium (Thermo Fisher Scientific). Immunoreactive images were captured using a BX-50 microscope (Olympus, Tokyo, Japan) equipped with a DP21 digital camera (Olympus).

## Supplementary Materials

Supplementary materials can be found at https://www.mdpi.com/1422-0067/20/24/6312/s1.

## Abbreviations

ER	Estrogen receptor
ERα	Estrogen receptor α
ERβ	Estrogen receptor β
ERE	Estrogen response element
E_2_	17β-estradiol
EtOH	Ethanol
DAPI	4,6-diamino-2-phenylindole
AVPV	Anteroventral paraventricular nucleus
PVH	Paraventricular nucleus of hypothalamus
UTR	Untranslated region
RACE	Rapid amplification of cDNA end
ORF	Open reading frame
FBS	Fetal bovine serum
DMEM	Dulbecco’s modified Eagle medium
PBS	Phosphate-buffed saline
PB	Phosphate buffer
TBS	Tris-buffered saline
BSA	Bovine serum albumin

## References

[1] Nilsson S., Gustafsson J.A., Bunce C., Campbell M. (2010). Estrogen receptors: Their actions and functional roles in health and disease. Nuclear Receptors, Proteins and Cell Regulation.

[2] Nuclear Receptors Nomenclature Committee (1999). A unified nomenclature system for the nuclear receptor superfamily. Cell.

[3] Hirata S., Shoda T., Kato J., Hoshi K. (2003). Isoform/variant mRNAs for sex steroid hormone receptors in humans. Trends Endocrinol. Metab..

[4] Kos M., Reid G., Denger S., Gannon F. (2001). Minireview: Genomic organization of the human ERalpha gene promoter region. Mol. Endocrinol..

[5] Hattori Y., Ishii H., Morita A., Sakuma Y., Ozawa H. (2015). Characterization of the fundamental properties of the N-terminal truncation (Δ exon 1) variant of estrogen receptor α in the rat. Gene.

[6] Ishii H., Kobayashi M., Munetomo A., Miyamoto T., Sakuma Y. (2013). Novel splicing events and post-transcriptional regulation of human estrogen receptor α E isoforms. J. Steroid Biochem. Mol. Biol..

[7] Ishii H., Sakuma Y. (2011). Complex organization of the 5′-untranslated region of the mouse estrogen receptor α gene: Identification of numerous mRNA transcripts with distinct 5′-ends. J. Steroid Biochem. Mol. Biol..

[8] Kobayashi M., Ishii H., Sakuma Y. (2011). Identification of novel splicing events and post-transcriptional regulation of human estrogen receptor α F isoforms. Mol. Cell. Endocrinol..

[9] Ishii H., Kobayashi M., Sakuma Y. (2010). Alternative promoter usage and alternative splicing of the rat estrogen receptor alpha gene generate numerous mRNA variants with distinct 5′-ends. J. Steroid Biochem. Mol. Biol..

[10] Lee M.T., Ouyang B., Ho S.M., Leung Y.K. (2013). Differential expression of estrogen receptor beta isoforms in prostate cancer through interplay between transcriptional and translational regulation. Mol. Cell. Endocrinol..

[11] Smith L., Brannan R.A., Hanby A.M., Shaaban A.M., Verghese E.T., Peter M.B., Pollock S., Satheesha S., Szynkiewicz M., Speirs V. (2010). Differential regulation of oestrogen receptor β isoforms by 5’ untranslated regions in cancer. J. Cell. Mol. Med..

[12] Smith L., Coleman L.J., Cummings M., Satheesha S., Shaw S.O., Speirs V., Hughes T.A. (2010). Expression of oestrogen receptor beta isoforms is regulated by transcriptional and post-transcriptional mechanisms. Biochem. J..

[13] Iwamoto H., Hirata S., Shoda T., Kato J., Hoshi K. (2003). The novel 5’-untranslated first exon, exon 0H, of the rat estrogen receptor beta gene. Endocr. Res..

[14] Hirata S., Shoda T., Kato J., Hoshi K. (2001). The multiple untranslated first exons system of the human estrogen receptor beta (ER beta) gene. J. Steroid Biochem. Mol. Biol..

[15] O’Brien M.L., Park K., In Y., Park-Sarge O.K. (1999). Characterization of estrogen receptor-beta (ERbeta) messenger ribonucleic acid and protein expression in rat granulosa cells. Endocrinology.

[16] Kuiper G.G., Enmark E., Pelto-Huikko M., Nilsson S., Gustafsson J.A. (1996). Cloning of a novel receptor expressed in rat prostate and ovary. Proc. Natl. Acad. Sci. USA.

[17] Kuiper G.G., Carlsson B., Grandien K., Enmark E., Häggblad J., Nilsson S., Gustafsson J.A. (1997). Comparison of the ligand binding specificity and transcript tissue distribution of estrogen receptors alpha and beta. Endocrinology.

[18] Mosselman S., Polman J., Dijkema R. (1996). ER beta: Identification and characterization of a novel human estrogen receptor. FEBS Lett..

[19] Moore J.T., McKee D.D., Slentz-Kesler K., Moore L.B., Jones S.A., Horne E.L., Su J.L., Kliewer S.A., Lehmann J.M., Willson T.M. (1998). Cloning and characterization of human estrogen receptor beta isoforms. Biochem. Biophys. Res. Commun..

[20] Van Pelt A.M., de Rooij D.G., van der Burg B., van der Saag P.T., Gustafsson J.A., Kuiper G.G. (1999). Ontogeny of estrogen receptor-beta expression in rat testis. Endocrinology.

[21] Shughrue P.J., Lane M.V., Scrimo P.J., Merchenthaler I. (1998). Comparative distribution of estrogen receptor-alpha (ER-alpha) and beta (ER-beta) mRNA in the rat pituitary, gonad, and reproductive tract. Steroids.

[22] Saunders P.T., Fisher J.S., Sharpe R.M., Millar M.R. (1998). Expression of oestrogen receptor beta (ER beta) occurs in multiple cell types, including some germ cells, in the rat testis. J. Endocrinol..

[23] Saunders P.T., Maguire S.M., Gaughan J., Millar M.R. (1997). Expression of oestrogen receptor beta (ER beta) in multiple rat tissues visualised by immunohistochemistry. J. Endocrinol..

[24] Snyder M.A., Smejkalova T., Forlano P.M., Woolley C.S. (2010). Multiple ERbeta antisera label in ERbeta knockout and null mouse tissues. J. Neurosci. Methods.

[25] Skliris G.P., Parkes A.T., Limer J.L., Burdall S.E., Carder P.J., Speirs V. (2002). Evaluation of seven oestrogen receptor beta antibodies for immunohistochemistry, western blotting, and flow cytometry in human breast tissue. J. Pathol..

[26] Weitsman G.E., Skliris G., Ung K., Peng B., Younes M., Watson P.H., Murphy L.C. (2006). Assessment of multiple different estrogen receptor-beta antibodies for their ability to immunoprecipitate under chromatin immunoprecipitation conditions. Breast Cancer Res. Treat..

[27] Andersson S., Sundberg M., Pristovsek N., Ibrahim A., Jonsson P., Katona B., Clausson C.M., Zieba A., Ramström M., Söderberg O. (2017). Insufficient antibody validation challenges oestrogen receptor beta research. Nat. Commun..

[28] Hawse J.R., Carter J.M., Aspros K.G.M., Bruinsma E.S., Koepplin J.W., Negron V., Subramaniam M., Ingle J.N., Rech K.L., Goetz M.P. (2019). Optimized immunohistochemical detection of estrogen receptor beta using two validated monoclonal antibodies confirms its expression in normal and malignant breast tissues. Breast Cancer Res. Treat..

[29] Leygue E., Dotzlaw H., Lu B., Glor C., Peter H., Watson P.H., Murphy L.C. (1998). Estrogen receptor beta: Mine is longer than yours?. J. Clin. Endocrinol. Metab..

[30] Petersen D.N., Tkalcevic G.T., Koza-Taylor P.H., Turi T.G., Brown T.A. (1998). Identification of estrogen receptor beta2, a functional variant of estrogen receptor beta expressed in normal rat tissues. Endocrinology.

[31] Lu B., Leygue E., Dotzlaw H., Murphy L.J., Murphy L.C., Watson P.H. (1998). Estrogen receptor-beta mRNA variants in human and murine tissues. Mol. Cell. Endocrinol..

[32] Chu S., Fuller P.J. (1997). Identification of a splice variant of the rat estrogen receptor beta gene. Mol. Cell. Endocrinol..

[33] Yang S.H., Liu R., Perez E.J., Wen Y., Stevens S.M., Valencia T., Brun-Zinkernagel A.M., Prokai L., Will Y., Dykens J. (2004). Mitochondrial localization of estrogen receptor beta. Proc. Natl. Acad. Sci. USA.

[34] Gilad L.A., Schwartz B. (2007). Association of estrogen receptor beta with plasma-membrane caveola components: Implication in control of vitamin D receptor. J. Mol. Endocrinol..

[35] Hattori Y., Ishii H., Munetomo A., Watanabe H., Morita A., Sakuma Y., Ozawa H. (2016). Human C-terminally truncated ERα variants resulting from the use of alternative exons in the ligand-binding domain. Mol. Cell. Endocrinol..

[36] Ishii H., Hattori Y., Munetomo A., Watanabe H., Sakuma Y., Ozawa H. (2017). Characterization of rodent constitutively active estrogen receptor α variants and their constitutive transactivation mechanisms. Gen. Comp. Endocrinol..

