# Supplementary material for: Applicability of Anti-Human Estrogen Receptor β Antibody PPZ0506 for the Immunodetection of Rodent Estrogen Receptor β Proteins
Source: Int J Mol Sci. 2019 Dec 13;20(24):6312. doi: 10.3390/ijms20246312 (PMC6941125; doi:10.3390/ijms20246312)
Supplement: Supplementary file 1 [file ijms-20-06312-s001.pdf]

# Supplementary Materials

## Materials and Methods

pcDNA3.1-mER$\alpha$66 and pcDNA3.1-rER$\alpha$66 vectors were constructed in our previous study [5]. Firefly luciferase reporter vectors (pERE-Luc and pControl-Luc) were purchased from Thermo Fisher Scientific. A *Renilla* luciferase vector (pRL-TK) was obtained from Promega.

## Dual luciferase reporter assay

HEK293 cells were seeded on 48-well culture plates (Techno Plastic Products). For assays to evaluate the transactivation abilities of respective constructs, cells were transfected with 125 ng/well reporter vectors (pERE-Luc or pControl-Luc), 100 ng/well respective constructs, and 12.5 ng/well pRL-TK vector. For competitive assays to evaluate the suppressive effects of ER$\beta_{ins}$ variants, cells were transfected with 125 ng/well pERE-Luc vector, 25 ng/well ER$\alpha$66, ER$\beta$1, or corresponding empty constructs, 50 ng/well ER$\beta_{ins}$ or pCMV-Tag 2B empty vectors, and 12.5 ng/well of pRL-TK vector. Twenty-four hours after transfection, the cells were treated with 10 nM E2 or 0.1% EtOH in DMEM with 2.5% charcoal-stripped FBS and 1% penicillin/streptomycin solution for 24 h. The cells were then washed with PBS and lysed in 80 μL of 1 × Passive Lysis Buffer (Promega). Firefly and *Renilla* luciferase activities were measured using Dual Luciferase Reporter Systems (Promega) and a Lumat 9507 Luminometer (Berthold Technologies, Wildbad, Germany). Measurements were performed in duplicate. Relative luciferase activities were calculated as ratios of firefly/*Renilla* luciferase activities and normalized against respective mean values of empty vector-transfected and vehicle-treated samples.

## Statistical analysis

Data were expressed as the mean ± SEM of six separate experiments. Statistical differences were evaluated by Student's *t*-test or two-way AOVA followed by Tukey's test and Student's *t*-test. *P*-values below 0.05 were regarded as statistically significant.

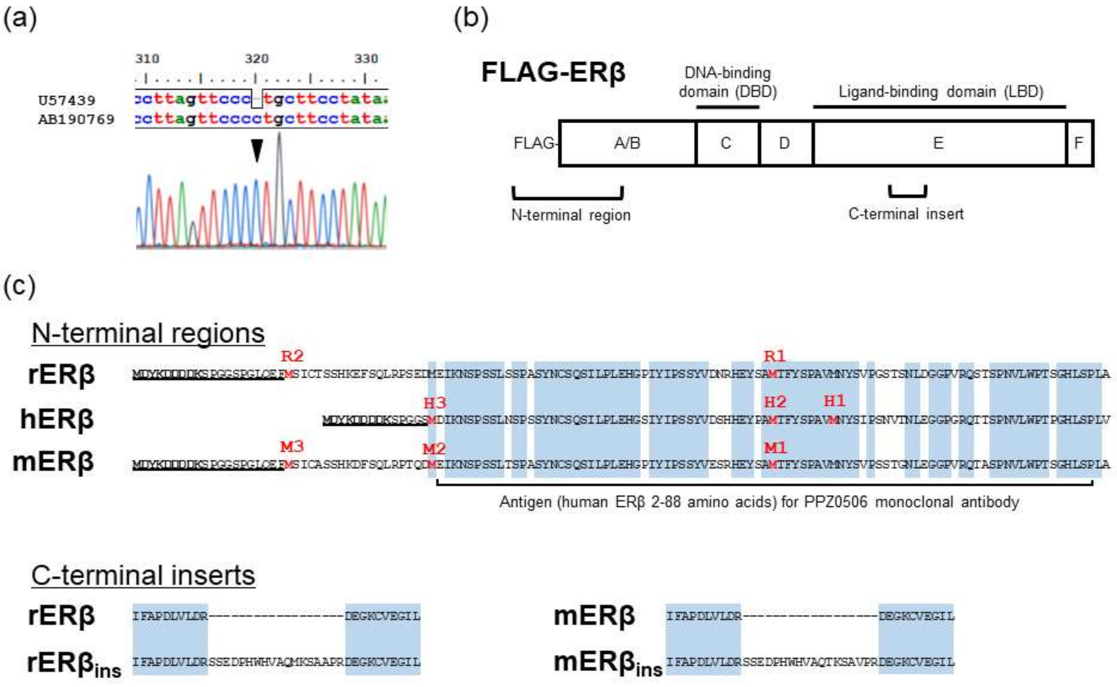


**Figure 1.** Sequences and structures of ERβ constructs. Nucleotide and amino acid sequences and schematic structures of ERβ constructs are shown. (a) An extra nucleotide insertion in the 5′-region of

the rat ERβ sequence. Pairwise alignment of U57439 and AB190769 sequences and the corresponding electropherogram of the cloned rat ERβ sequence are shown. (b) Schematic structure of FLAG-tagged ERβ proteins encoded in expression constructs. (c) Detailed amino acid sequences of the N-terminal regions of human, mouse, and rat ERβ proteins, and the inserted peptides of mouse and rat $ER\beta_{ins}$ proteins. Panel c was constructed with reference to Leygue et al. [28].

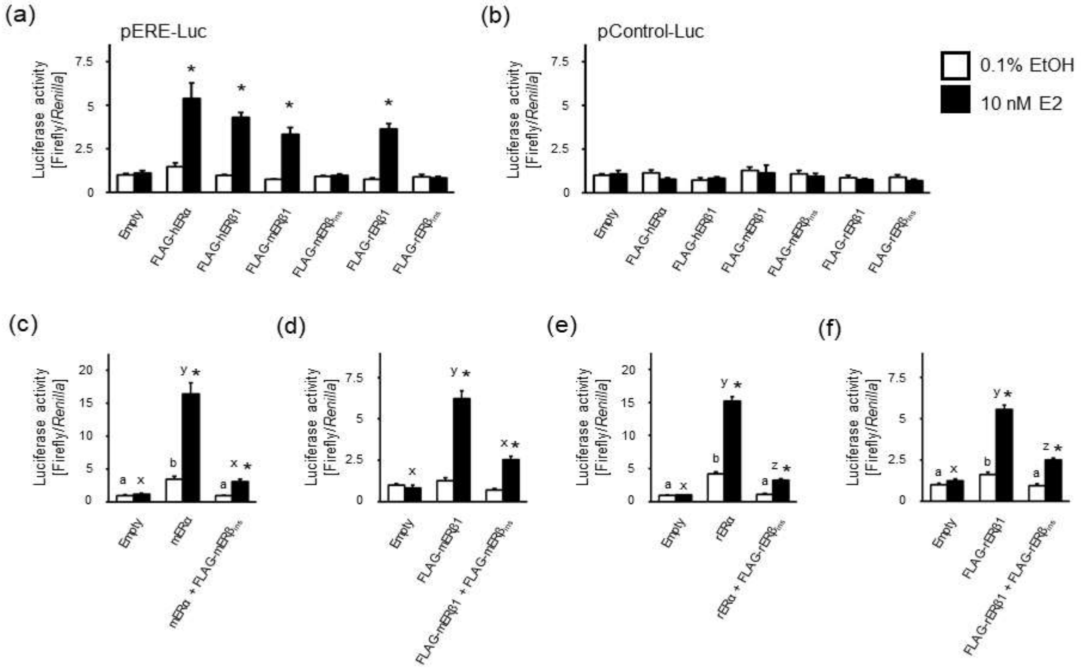


**Figure 2.** Transactivation activities of ERβ constructs. Functional expressions of human, mouse, and rat ERβ constructs in transfected cells were assessed by ERE luciferase reporter assays. (a) Transactivation of an ERE-driven promoter by human, mouse, and rat ERβ constructs. (b) Transactivation of an ERE-less minimum promoter by human, mouse, and rat ERβ constructs. (c-f) Repression of wild-type ER$\alpha$ (ER$\alpha$66; c and e)- and ERβ (ERβ1; d and f)-mediated transactivation by mouse (c and d) and rat (e and f) $ER\beta_{ins}$ constructs. Transfected cells were treated with 0.1% EtOH or 10 nM E2. Relative luciferase activities were calculated as ratios of firefly/*Renilla* luciferase activities and normalized against the respective mean values of the empty vector(s)-transfected and EtOH-treated samples. Six separate assays were performed (n = 6). Student's *t*-test: *, $P < 0.05$ between EtOH- and E2-treated groups; Tukey's test: different letter labeling (a-b and x-z), $P < 0.05$ among EtOH- and E2-treated groups, respectively.

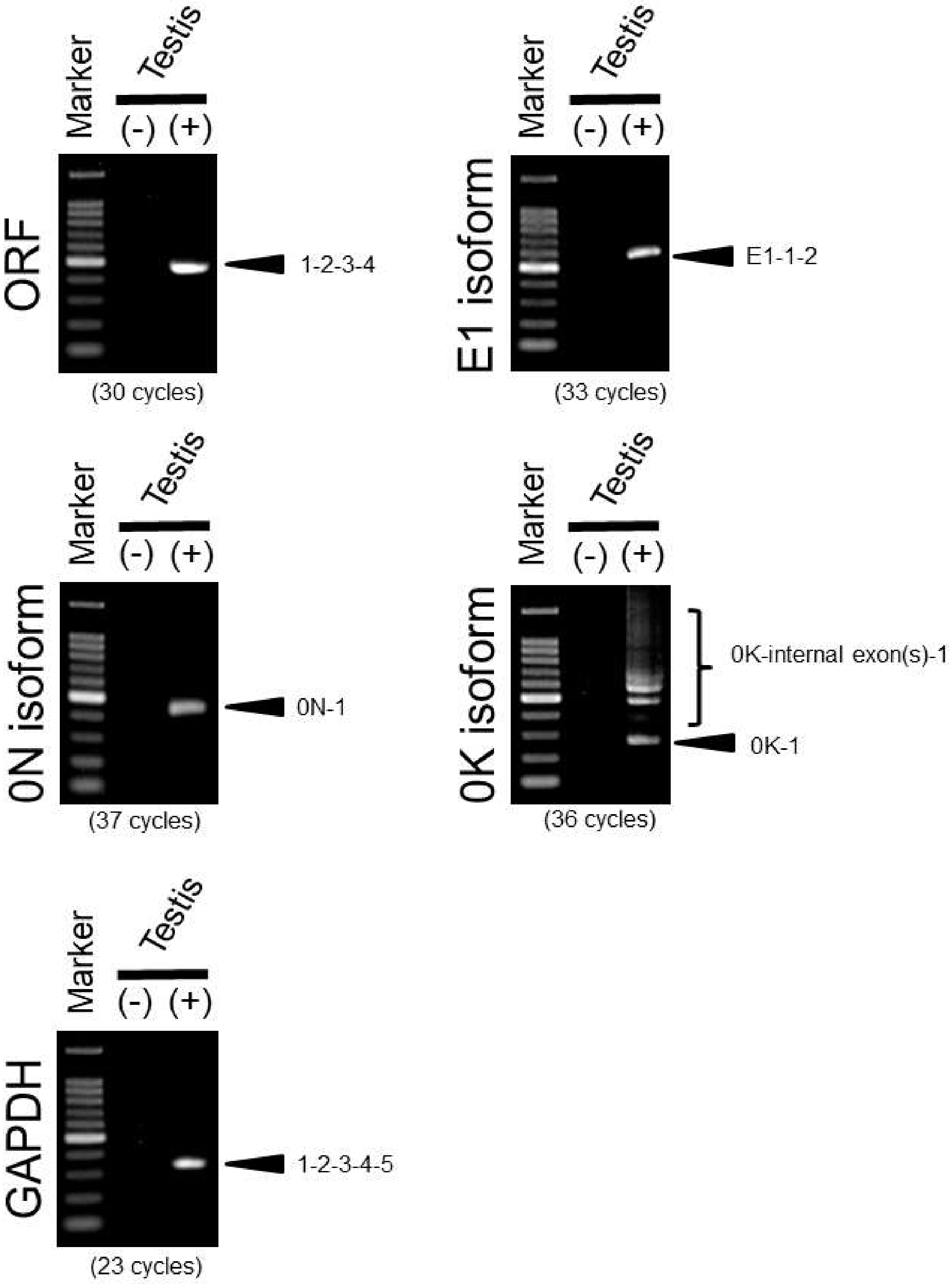


**Figure 3.** Alternative promoter usage and alternative splicing profiles of human ERβ gene in testis. The expression and splicing profiles of human ERβ isoforms in testis was investigated using RT-PCR. (−), Total RNA without reverse transcriptase; (+), reverse-transcribed cDNA.

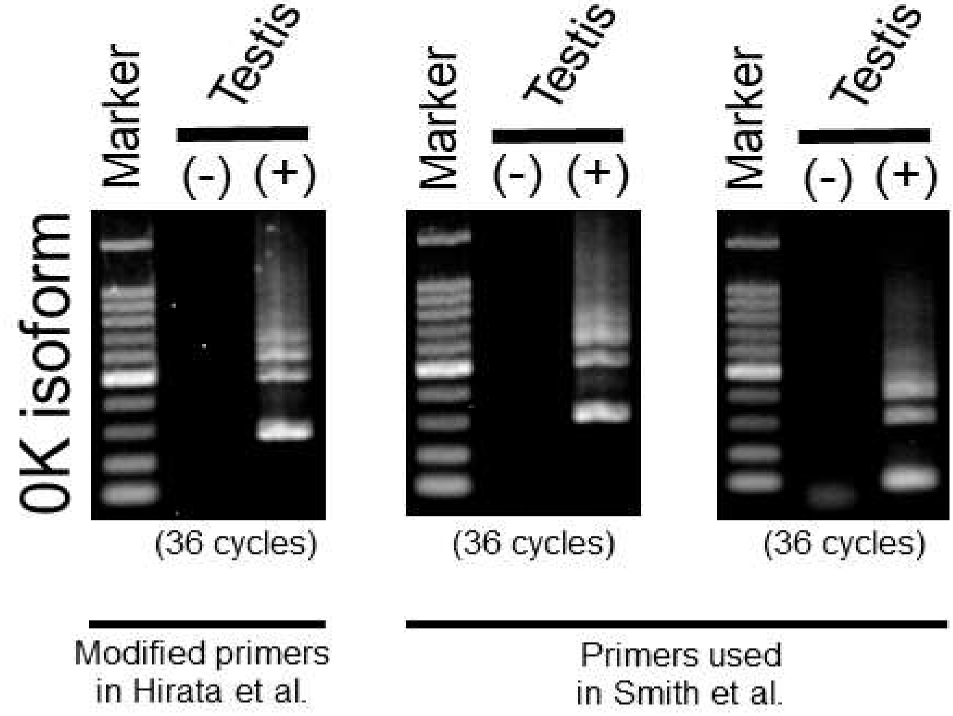


**Figure 4.** RT-PCR analysis of human ERβ 0K isoforms using primer pairs reported in previous studies. The expression and splicing profiles of human ERβ 0K isoforms in the testis was investigated using RT-PCR with primer pairs reported by Hirata et al. [14] and Smith et al. [11,12]. The band patterns observed using the primer pairs from previous studies are similar to those using our primer pairs (Suppl. Fig. 3). (−), Total RNA without reverse transcriptase; (+), reverse-transcribed cDNA.

(a) human exon 0K

```
GGCGGCGAGT CTCCCGGATG CTCCTCAGCT CTGGGGACGC GGTGCAGAAG
TGTGAGGGCG CCCGGCTTCC AGGCAGTAAT GGGCGGGTCC CTGCGCGGGA
GCGTGGCGGG CGCTGGACTC TACAGCAGAT GTGGAACTGG AGAGCTTGGC
GCGCCTTCCG ACTTTGTCAC ACACCTGCGC CGCCAGACTG GGGTCGGGCC
CCTCCGCGTT CTGCTCTGGA GTGCCTGGGT CTGGGCCCAG CACCGCGCTT
TTAGAATCTC CTCAGCTGAA TCTGACGCTC AGCAGTGGGT GAAGCGCAGC
CCCCTGTTTC AGGCCCTGCC GAGCTGGAAG GAGTGTCAGA GCTGGAGCGC
GCGTGGCCCC CTCTGTGTTG GGGTCACCCC GGGGTTGCCA GGGCTCAGGG
AGGGTCGTAG TCTGGATTTT GTCACCCGCA CGTCCCCACC CCCCAGCAGG
TCTGGGGTTG GAGAATCCAC GCGGGCTTCA TAAGCTAGAT GCCAGTTAAC
TGTCGAGAGG GGACGCTCCC TCCTCGTAGG CGTCCACACT GGAGAAGGAA
TAAGATGGGC GATTGCCTGG GAAGCCTGAC AGGGCGGCGG CAGCTGGGAT
GCTGGAGAGG ACTGGCCCCT TGAGTTACTG AGTCCGATGA ATGTGCTTGC
TCTGCTGGAG GAACCGCGCT CAGGTTACAG TCATCCCAAT ATGGTTCTGA
AG
```

(b) human exon 0X1

```
TTCTCAAAGT TTGCACAAGC GGATATTTTA GAGGTACAGT GTAATATAAG
AGCTTCTGAA AATGTCCACT TAAGTTGTTT TATACCTGAG CAAGTGAAAT
TAAGAAGGGA ATTGAAGCAA ATATTCCTG
```

(c) human exon 0X2

ACATCCAAGT GGAGATATGG CATTTAAATT CATGAGATTG GATGAGATCC
CACCAAAGGA ACAGGTTTAG GTGGAGACAA CCAAATACCG ATGCCTAGGA
CACTGCAGTG TTTAGAATTC AAGGAGATGA GAAGGAAACA GGAGGGAAGA
TTGAAAAGAA GAGTCCAGTG TGTTATGAGG AAAACCCCAA GAGCATGCTG

CCTTACAAGA CAGGTGAAAA ATGTGTTCTG TGAAAGAAAG AGTAATTAAC
TGTTAAATGT TACAGACTGA TCAAATAAAA TGAAGACTGA GAATGGCCTG
TTTGTAAG

(d) human exon 0X3

ATCACTTTTA AAAGGAAAAC ATAGGAGCCT GAAACAGAAG TGGGAAACAA
ATATTTACTC AAACTAAGAG ACTAAACTCA GTAGCCAGCA ACAAGAGATC
AAG

(e) human exon 0X4

ATGGAGTCCT CCTCTGTCAC CCAGGCTGGA ACGCAGTGGT ATGATCTCGG
CTAACTGCAA CCTCAGCCTG CCAGGTTCAA GCAATTCTTC TGCCTCAGCC

TCCCGAGTAG CTGGGATTAC AGGTGCCTGC TGCCATGATG ATTAATTTTA
TGTGTTAACT TAGCTGGGCT GTGTTGCCCA GATAGTTGGT TAAACATTAT
TCTGGATGTT TCTGTGAAGA TGTTTTTGGA TGAGGTTAAC ATTTAGATCG
GTGGACTTTG AGTAAAGCAG ATTACCTTTC ATAATTTGGG TGGGGCTCAT
CCAATCAGTT GAACATCTGA AGAGACCAAA AGACTGACCT TCTGCAAGCA
AAGAAAAATT CTGCCAACAG ACAGCCATTG GACTTGAACT TCAACATTGA
CTCTTCAGTC TATTGGCCCA CCCTGCAAAT TTTGGACTTG CCA

(f) human exon 0Y1

GGACTCTAGA AATGCCAGAT AATTCCACTT TTGTGGTGAC AGAAGAATCT
GGCAATAATA GCTACCGTTT ACTGAACAAC AACTGCACAT TAAGCACTGT
GTCATATGCT TTAG

(g) human exon 0X5

AGGGAGACAT CAACCTGTTG TGGAAAAGAA TGATCACTTA AAGTCTTTAG
AAATTCTGAA CCAACTCTCT AGCAGGTGAT CCTTGTTAGA ATTTGAGCCC
TTAACGCTAT CCAGGACTGG AGGTTGAAGG GACGATAGAG GGAGCAGGAG
GAGAATGCAC ATGGATTAAG GAGCGAGAAC ACAG

(h) human exon 0X6

AAATCCTGGG CTCTCTTCTC CCAGCCACAA GGTTAGGTTG AAAAACAGAG
CAGATGGAGG TAGTTTGTAG CCTACAGGTG CCCTGAATGA AGCTTCCACA
GTGCTAAAGT GGAAGAACGA GGGACTCCAA GGGAAGGATT CAAGGCTGGG
CCCATGCACC TGTGTAATTC AGAAGAGACC CCAGAGGAGA TCAGCGCCCT
CTAATTAGCC CTG

(i) human exon 0X7

TATCTGGGCT CTACAGGACA GACATGCCTC CATTTATGCA ACAAATAAGA
ACAGCATCTC ATGACAGTGG AGAAAACATG GGATGTGCAG GTAG

(j) human exon 0X8

GGTTTTGTTT TGCCTCTTGG TAGTTTCTTT CCTACGGAAA ATTCTCCCTC
TGATCTTTCC AAGTCAAAGG CTTCAGCAAA CATTTGTTGA ACGCGTGGAT
TGTGCTAGGT GGGTGTTATG GACCATGGAG AATGCTAGAG ATGTAAGACA

TGCGCTGTCC AATCGCAGCG CAGGTTGTGT TGACAG

(k) human exons 0N and 0Y2
GGCTCGGTCA CGTGGGCTCA GGCACTACTC CCCTCTACCC TCCTCTCGGT
CTTTAAAAGG AAGAAGGGGC TTATCGTTAA GTCGCTTGTG ATCTTTTCAG
TTTCTCCAGC TGCTGGCTTT TTGGACACCC ACTCCCCCGC CAGGAGGCAG
TTGCAAGCGC GGAGGCTGCG AGAAATAACT GCCTCTTGAA ACTTGCAGGG
CGAAGAGCAG GCGGCGAGCG CTGGGCCGGG GAGGGACCAC CCGAGCTGCG
ACGGGCTCTG GGGCTGCGGG GCAGGGCTGG CGCCCGGAGC CTGAGCTGCA
GGAGGTGCGC TCGCTTTCCT CAACAGGTGG CGGCGGGGCG CGCGCCGGGA
GACCCCCCCT AATGCGGGAA AAGCACGTGT CCGCATTTTA GAGAAGGCAA
GGCCGGTGTG TTTATCTGCA AG

(l) human exons E1 and 1
ATTTTCATGT ATATTTTTCA GGATGTATTT GTAATCTCAT ACAAACGTAT
GTATTTTTTT AATGAAAATA TTTAAATTTT CATAGTTAAC AGCTGTAGCT
CTAACTTGGC AATATCTTCT GTGTTTCTTT ACAGCCATTA TACTTGCCCA
CGAATCTTTG AGAACATTAT AATGACCTTT GTGCCTCTTC TTGCAAGGTG
TTTTCTCAGC TGTTATCTCA AGACATGGAT ATAAAAAACT CACCATCTAG
CCTTAATTCT CCTTCCTCCT ACAACTGCAG TCAATCCATC TTACCCCTGG
AGCACGGCTC CATATACATA CCTTCCTCCT ATGTAGACAG CCACCATGAA
TATCCAGCCA TGACATTCTA TAGCCCTGCT GTGATGAATT ACAGCATTCC
CAGCAATGTC ACTAACTTGG AAGGTGGGCC TGGTCGGCAG ACCACAAGCC
CAAATGTGTT GTGGCCAACA CCTGGGCACC TTTCTCCTTT AGTGGTCCAT
CGCCAGTTAT CACATCTGTA TGCGGAACCT CAAAAGAGTC CCTGGTGTGA
AGCAAGATCG CTAGAACACA CCTTACCTGT AAACAG

(m) rat exon 0N/P1
ACACTCTTTT CTAGGTCTTT AAAAGACGCA CTAACATCCG TTAGTCGTGG
GTAATCTTTG CAGCTTCTCC AGCTGCTGGC CTTTTTGAAA CGCACTCTCA
GGTCCCTGCC TTCAGCGAGG CTTCTAGAAT CAGCCACCTC TTGAAACTTC
TTGGTGGGGA GCTGGCCCAG GGGGAGCGGC TGGTGCTGCC ACTGGCATCC
CTAGGCACCC AGGTCTGCAA TAAAGTCTGG CAGCCACTGC ATGGCTGAGC
GACAACCAGT GGCTGGGAGT CCGGCTCTGT GGCTGAGGAA AGCACCTGTC
TGCATTTAGA GAATGCAAAA TAGAGAATGT TTACCTGCCA G

(n) rat exons E1/P2 and 1
TGATTATATG GAAGCCCCAT TGCCCCTAGC TAAAATGAAT ATGTCTTAGT
CACTCTGGCA GCTTGAACTA ACCAGACATC GTTTGCTTTC CTCTGCAGTC
ATTACATCTG AGTCCCATGA GTCTCTGAGA ACATAATGTC CATCTGTACC
TCTTCTCACA AGGAGTTTTC TCAGCTGCGA CCCTCTGAAG ACATGGAGAT
CAAAAACTCA CCGTCGAGCC TTAGTTCCCC TGCTTCCTAT AACTGTAGCC
AGTCCATCCT ACCCCTGGAG CACGGCCCCA TCTACATCCC TTCCTCCTAC
GTAGACAACC GCCATGAGTA TTCAGCTATG ACATTCTACA GTCCTGCTGT
GATGAACTAC AGTGTTCCCG GCAGCACCAG TAACCTGGAC GGTGGGCCTG
TCCGACAGAG CACAAGCCCA AATGTGCTAT GGCCAACTTC TGGGCACCTG
TCTCCTTTAG CGACCCATTG CCAATCATCG CTCCTCTATG CAGAACCTCA
AAAGAGTCCT TGGTGTGAAG CAAGATCACT AGAGCACACC TTACCTGTAA
ACAG

**Figure 5.** Nucleotide sequences of 5′-UTR exons of human and rat ERβ genes. Nucleotide sequences of untranslated leader exons (0K, 0N, and E1) and internal exons (0X1-8 and 0Y1-2) in the 5′-regions of human and rat ERβ genes are shown. Alternative splicing donor and acceptor sites are indicated by open and closed arrowheads, respectively. The GT/AG boundaries of the alternative splice sites are underlined. The shaded region in human exon 0X7 refers to Moore et al. [19], Lee et al. [10], and sequences AB006589 and KC777387, and the shaded regions in rat exons 0N/P1 and E1/P2 refer to O′Brien et al. [15]. Of note, the human exon 0X5 sequence (KC777385) registered by Lee et al. [10] is in the antisense direction.

**Table 1.** Clones identified in 5'-RACE and RT-PCR experiments.

| Organ | Experiments | Targets | Clones |
|---|---|---|---|
| Testis | 5'-RACE | 5'-UTR variants | 0K-1, 0K-0X1-1, 0K-0X2$_L$-1, 0K-0X2$_S$-1, 0K-0X1-0X2$_L$-1, 0K-0X1-0X2$_S$-1, 0K-0X2$_S$-0X4$_L$-1, 0K-0X6-0X7$_S$-1, 0K-0X2$_S$-0X4$_S$-0Y1-0X5-1, 0K-0X2$_S$-0Y1-0X5-0X6-0X7$_S$-0X8-1, 0N-1, E1-1 |
| | RT-PCR | ORF | 1-2-3-4 |
| | | 0K isofrms | 0K-1, 0K-0X1-1, 0K-0X2$_L$-1, 0K-0X2$_S$-1, 0K-0X4$_S$-1, 0K-0Y2-1, 0K-0X1-0X2$_L$-1, 0K-0X1-0X2$_S$-1, 0K-0X2$_L$-0X4$_L$-1, 0K-0X2$_S$-0X4$_L$-1, 0K-0X2$_S$-0X5-1, 0K-0X2$_S$-0X6-1, 0K-0X2$_S$-0X7$_S$-1, 0K-0X6-0X7$_S$-1, 0K-0X2$_L$-0X3-0X4$_S$-1, 0K-0X2$_S$-0Y1-0X5-1, 0K-0X2$_S$-0X5-0X6-1, 0K-0X2$_S$-0X4$_S$-0Y1-0X5-1,0K-0X2$_S$-0Y1-0X5-0X6-0X7$_S$-0X8-1 |
| | | 0N isoforms | 0N-1 |
| | | E1 isoforms | E1-1-2 |
| Ovary | 5'-RACE | 5'-UTR variants | 0N/P1-1, E1/P2-1 |
| Prostate | | | 0N/P1-1 |
| Ovary/Prostate | RT-PCR | ORF | 7-8 |
| Ovary/Prostate | | 0N/P1 isoform | 0N/P1-1 |
| Ovary | | E1/P2 isoforms | E1/P2-1-2 |

**Table 2.** Oligonucleotide primers used for 5'-RACE and RT-PCR.

| Purpose | Species | Gene | Exon | Direction | Oligonucleotide sequence (5' to 3') | Comment/Reference |
|---|---|---|---|---|---|---|
| 5'-RACE | | Universal | | Forward | 5'-AAGCAGTGGTATCAACGCAGAGTACXXXXX-3' | 5'-RACE adapter primer |
| | | | | Forward | 5'-CTAATACGACTCACTATAGGGCAAGCAGTGGTATCAACGCAGAGT-3' | 5'-RACE universal primer mix |
| | | | | Forward | 5'-CTAATACGACTCACTATAGGGC-3' | 5'-RACE universal primer mix |
| | | | | Forward | 5'-AAGCAGTGGTATCAACGCAGAGT-3' | 5'-RACE nested universal primer |
| | Human | *ESR2* | 1 | Reverse | 5'-GTTTACAGGTAAGGT-3' | 5'-RACE gene-specific RT primer |
| | | | 1 | Reverse | 5'-AACACATTTGGGCTTGTGGT-3' | 5'-RACE gene-specific primer |
| | | | 1 | Reverse | 5'-TCCAGGGGTAAGATGGATTG-3' | 5'-RACE gene-specific primer |
| | Rat | *Esr2* | 2 | Reverse | 5'-TAATGATACCCAGAT-3' | 5'-RACE gene-specific RT primer |
| | | | 1 | Reverse | 5'-CAATGGGTCGCTAAAGGAGA-3' | 5'-RACE gene-specific primer |
| | | | 1 | Reverse | 5'-TAAGGCTCGACGGTGAGTTT-3' | 5'-RACE gene-specific primer |
| RT-PCR | Human | *ESR2* | 1 | Forward | 5'-CACCTGGGCACCTTTCTCCTTTAG-3' | NM_001437 |
| | | | 4 | Reverse | 5'-GCTCGTCGGCACTTCTCTGTCTC-3' | NM_001437 |
| | | | 0K | Forward | 5'-TGGCCCCTTGAGTTACTGAG-3' | BX457807 |
| | | | 1 | Reverse | 5'-TCCAGGGGTAAGATGGATTG-3' | NM_001437 |
| | | | 0K | Forward | 5'-CGATTGCCTGGGAAGCC-3' | Ref. [11] |
| | | | 1 | Reverse | 5'-AGGAAGGTATGTATATGGAGCCG-3' | Ref. [11] |
| | | | 0K | Forward | 5'-AGTTACTGAGTCCGATGAATGTGCTTG-3' | Ref. [12] |
| | | | 1 | Reverse | 5'-CTCAAAGATTCGTGGGCAAGTATAATG-3' | Ref. [12] |
| | | | 0K | Forward | 5'-GGAGGAACCGCGCTCAGGTTA-3' | Ref. [14] |
| | | | 1 | Reverse | 5'-GGCTATAGAATGTCATGGCTGG-3' | Ref. [14] |
| | | | 0N | Forward | 5'-AGGCTGCGAGAAATAACTGC-3' | NM_001437 |
| | | | 1 | Reverse | 5'-TCCAGGGGTAAGATGGATTG-3' | NM_001437 |
| | | | E1 | Forward | 5'-TAACAGCTGTAGCTCTAACTTG-3' | Ref. [12] |
| | | | 2 | Reverse | 5'-CATCCCTCTTTGAACCTGGA-3' | NM_001437 |
| | | *ESR1* | 2 | Forward | 5'-TCAGATAATCGACGCCAGGGTG-3' | NM_000125 |
| | | | 3 | Reverse | 5'-CACTTCGTAGCATTTGCGGAGCC-3' | NM_000125 |
| | | *GAPDH* | | Forward | 5'-TTCGACAGTCAGCCGCATCTTCTTTTG-3' | NM_002046 |
| | | | | Reverse | 5'-CGCCAGCATCGCCCCACTTG-3' | NM_002046 |
| | Rat | *Esr2* | 7 | Forward | 5'-GCAAACCAGGAGGCAGAAAGTAGC-3' | AB190769 |
| | | | 8 | Reverse | 5'-AAGTGGGCAAGGAGACAGAAAGTAAGTA-3' | AB190769 |
| | | | 0H | Forward | 5'-TTATCCTTCCTGACGGACAG-3' | Ref. [13] |
| | | | 1 | Reverse | 5'-TAAGGCTCGACGGTGAGTTT-3' | AB190769 |
| | | | 0N | Forward | 5'-AGGAAAGCACCTGTCTGCAT-3' | Ref. [15] |
| | | | 1 | Reverse | 5'-CAATGGGTCGCTAAAGGAGA-3' | AB190769 |
| | | | E1 | Forward | 5'-TTATATGGAAGCCCCATTGC-3' | Ref. [15] |
| | | | 2 | Reverse | 5'-CGCCGTAATGATACCCAGAT-3' | AB190769 |

| | | | | |
|---|---|---|---|---|
| *Esr1* | 6 | Forward | 5'-ACCTGCAGGGAGAAGAGTTTGTGT-3' | AB477039 |
| | 8 | Reverse | 5'-CTTGTGGGGAGCCTGGGAGTTC-3' | AB477039 |
| *Gapdh* | | Forward | 5'-TGTGCAGTGCCAGCCTCGTCTCATA-3' | NM_017008 |
| | | Reverse | 5'-ACCCTTTTGGCCCCACCCTTCAG-3' | NM_017008 |
